# Erythrocytes do not produce biologically active NO

**DOI:** 10.1186/2050-6511-16-S1-A53

**Published:** 2015-09-02

**Authors:** Stepan Gambaryan, Igor Mindukshev, Natalia Rukoyatkina, Iraida Sharina, Emil Martin, Ulrich Walter

**Affiliations:** 1 Sechenov Institute of Evolutionary Physiology and Biochemistry, Russian Academy of Sciences, St. Petersburg, Russia; 2 Institute of Clinical Biochemistry and Pathobiochemistry, University of Würzburg, Würzburg, Germany; 3 Department of Internal Medicine, Division of Cardiology, University of Texas Houston Medical School, Houston, TX, USA; 4 Center for Thrombosis and Hemostasis (CTH), Johannes Gutenberg University, Mainz, Germany

## 

The main function of red blood cells (RBC), which is mediated by Hb, is the transport of oxygen and carbon dioxide in the body. In addition, RBC are strongly involved in regulation of vascular tone especially under hypoxic conditions. Hypoxic vasodilation is a fundamental physiological process which directly correlates with the oxygen saturation of Hb in RBC. Since discovery in 1980s that NO is a biological signalling molecule, numerous studies linked NO/Hb to the mechanisms underlying hypoxic vasodilation. According to calculations, mathematical modelling, and experimental data most of NO in blood is scavenged by Hb by the reaction of deoxyHb with NO which results in formation of metHb and nitrate. It has been also proposed that NO can bind by nitrosylation reaction of conserved cysteine on Hb β-chain (β93Cystein) to form SNO-Hb. Although different reactions between NO and Hb are clear and well established, the mechanisms of NO release from Hb and how, in physiological conditions of high excess of free Hb, NO can escape from scavenging by Hb are not so well established. Importantly, till now, there was never shown directly that RBC released biologically active NO can activate soluble guanylyl cyclae (sGC) in neighboring cells (platelets or vascular smooth muscle cells).

Most experiments that prove NO release from RBC are done in blood vessel relaxation or in isolated washed RBC. However, both these experimental approaches are not sufficient to clearly answer the question whether RBC can produce biologically active NO because vasorelaxation is a complex reaction which is not mediated only by NO and in isolated RBC experiments it was never shown that RBC produced NO or other NO related radicals could activate sGC. To answer this question we used two different approaches which allow to directly measure NO-mediated activation of sGC in intact cells (platelets) and in purified enzyme. Endogenous NO is a well-known and potent inhibitor of platelet activation and platelet inhibitory effects of NO are mediated solely by sGC activation. Intact platelet sGC is highly sensitive to endogenous NO and even nanomolar NO concentrations are sufficient for cGMP generation. In platelets, the main mediator of cGMP is PKG which phosphorylates multiple substrates responsible for platelet inhibition. For evaluation of platelet sGC activity we used our well-established model based on NO/sGC/cGMP/PKG dependent vasodilator-stimulated phosphoprotein (VASP) phosphorylation in human platelets and purified sGC which is also very sensitive to low NO concentrations. In our experiments RBC were mixed with platelet suspension or with purified enzyme and sGC activation was assessed by VASP phosphorylation and cGMP formation. All our experiments clearly show that RBC, in any conditions, cannot be sources of NO, they act only as a strong NO scavengers and all our data, albeit indirectly, indicate that hypoxia/RBC induced vasodilation is not mediated by NO derived from RBC.

